# How to evaluate the use of GPS tracking devices to support “safer walking” for people with dementia: is a randomised controlled trial feasible?

**Published:** 2012-05-18

**Authors:** Heather Milne, Brian McKinstry

**Affiliations:** University of Edinburgh, UK; University of Edinburgh, UK

**Keywords:** telecare, randomised controlled trials, GPS tracking in dementia

## Abstract

**Introduction:**

Dementia costs the UK economy £23 billion each year, the majority of which is the cost of informal care and long-term institutional social care. One aspect of dementia which adds to carer stress and leads to earlier admission into long-term care is people with dementia becoming ‘lost’ or disorientated when out walking in the community. Many people with dementia feel compelled to go out walking, and while physical exercise has been associated with psychological and physical benefits, 40% of people with dementia get ‘lost’ outside at some point. Current recommendations are that “wandering” behaviour should be managed through non-pharmacological interventions such as exercise, multi-sensory environments and music therapy. Global positioning systems (GPS) for people with dementia are also becoming popular with private individuals and social care services to enable “safer walking”. The GPS tracking device carried by the person with dementia allows their carer to locate them quickly and easily if they become ‘lost’. Many of the devices also have a “geofence” feature which alerts the carer if the person moves outwith an agreed area. It is hoped that the use of GPS devices will support continued independence and access to the outdoor environment for the person with dementia while giving peace of mind to both them and their carer. Reviews of current research argue that the evidence is insufficiently robust to support any of the current non-pharmacological interventions for “wandering”, including the use of GPS tracking devices. Reviews have called for high quality studies, preferably randomised controlled trials (RCTs) to determine the clinical and cost effectiveness of these interventions. RCTs are considered the gold standard for informing evidence based policy in both health and social care, but they require the control of multiple variables, equivalent groups of users and controls, and valid measurable outcomes. However, these conditions may be difficult to achieve when evaluating interventions in complex health and social care situations.

**Aims and objectives:**

Our research aims to consider the feasibility of conducting an RCT into the effectiveness of GPS tracking devices for people with dementia who may become ‘lost’ outside their home. We are exploring service users’ and professionals’ experiences of using a GPS tracking device and whether its use is associated with changes in levels of carer stress over a six-month period. The study is also trialling an assessment of whether having a GPS influences health and social service usage in order to understand if it is feasible to calculate cost effectiveness. This presentation will outline preliminary findings from this research and reflect on what other methods might be employed to evaluate the GPS tracking intervention if an RCT proves not to be feasible.

